# Supplementary material for: Novel Highly Efficient Antibacterial Chitosan-Based Films
Source: BioTech (Basel). 2023 Jul 7;12(3):50. doi: 10.3390/biotech12030050 (PMC10366851; doi:10.3390/biotech12030050)
Supplement: Supplementary file 1 [file biotech-12-00050-s001.zip › biotech-2438279-supplementary.pdf]

Supplementary Materials

(A)

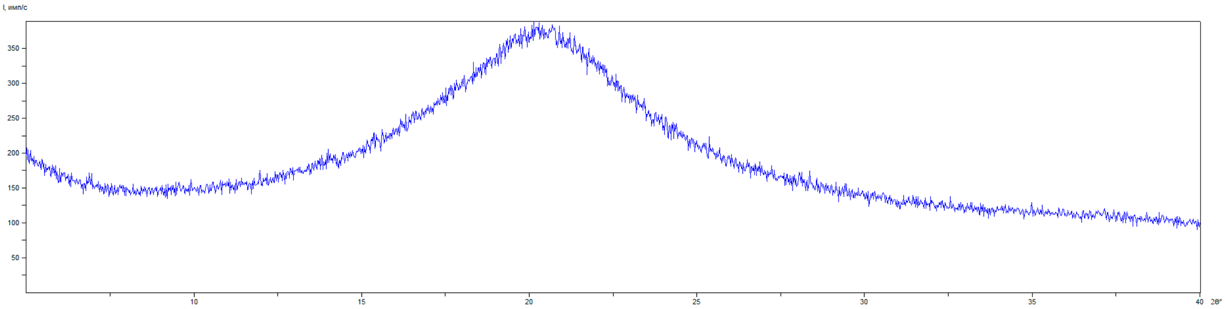

(B)

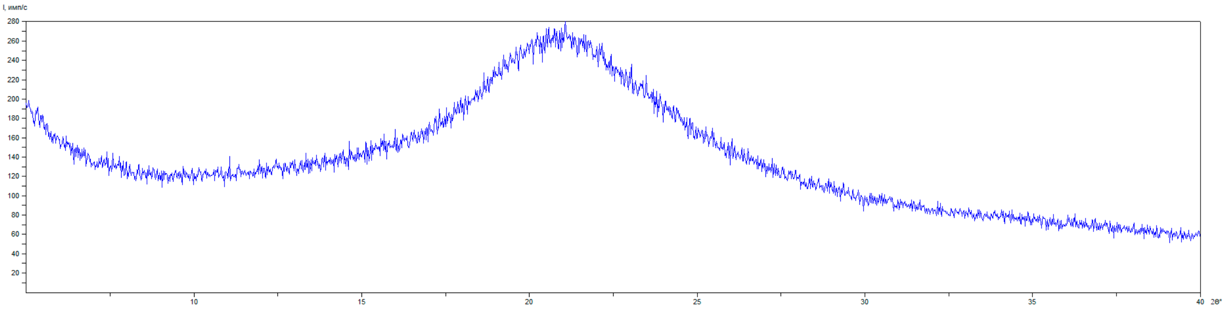

(C)

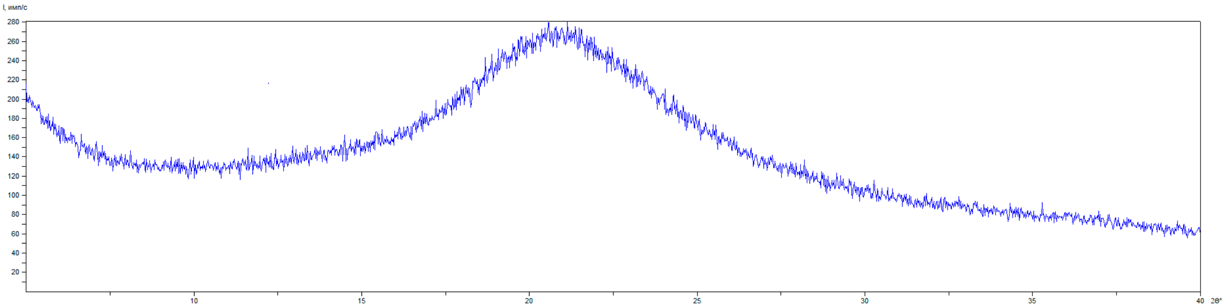

(D)

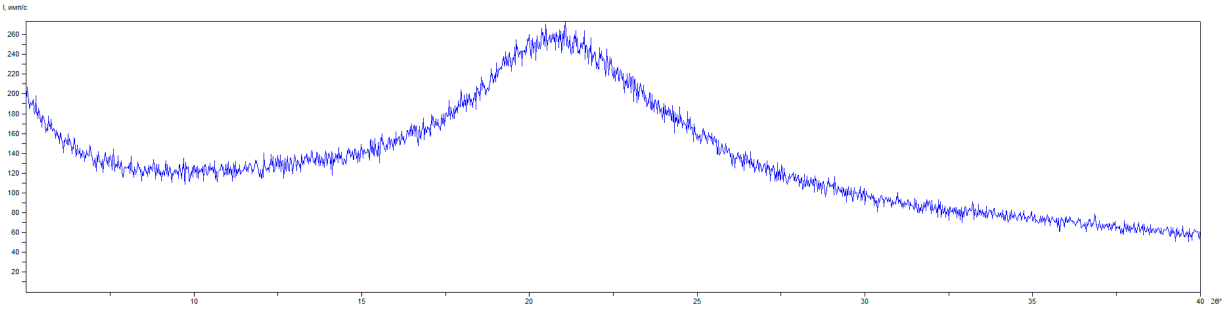

(E)

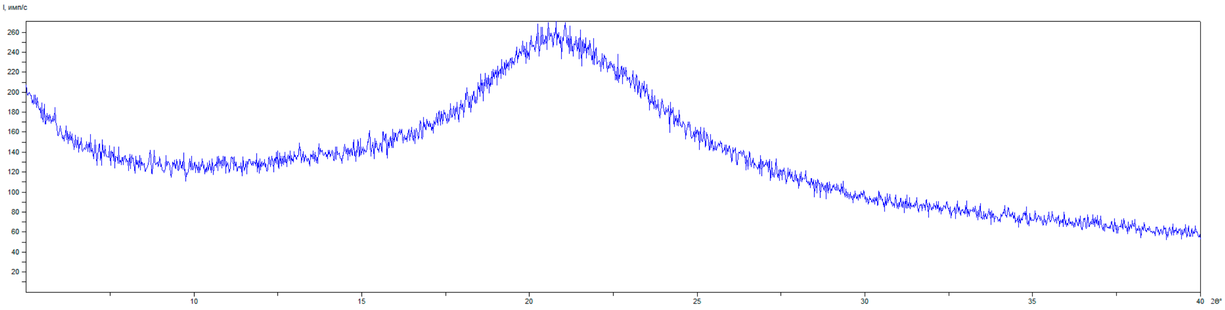

(F)

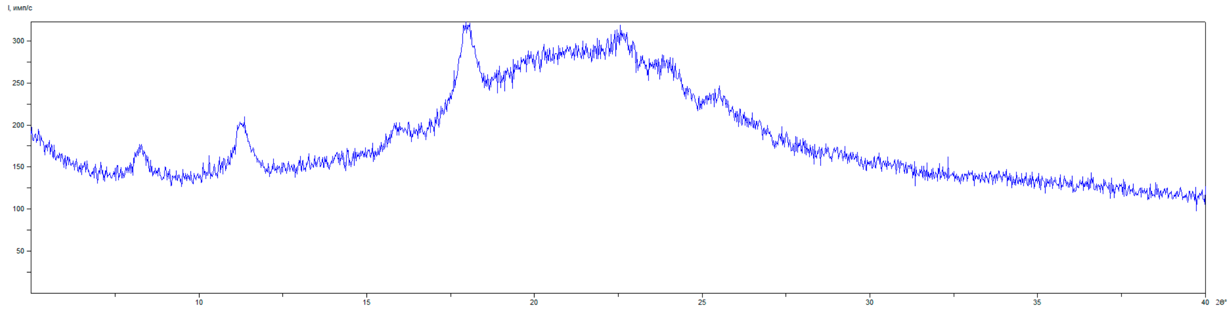

Figure S1. The X-ray diffraction patterns of films **O** (**A**); **A2** (**B**); **B5** (**C**); **C10** (**D**); **D15** (**E**) and **Q** (**F**).
